# The Effect of Oral Intake of Honey Syrup on the Pain Intensity of Active Phase of Parturition of nulliparous women: A Randomized clinical trial

**DOI:** 10.22088/cjim.10.1.98

**Published:** 2019

**Authors:** Simin Taavoni, Leila Fathi, Neda Nazem-Ekbatani, Hamid Haghani

**Affiliations:** 1 Research Institute for Islamic and Complementary Medicine, Iran University of Medical Sciences; Tehran, Iran; 2 School of Nursing and Midwifery, Lorestan University of Medical Sciences, Khoramabad, Iran; 3 School of Nursing and Midwifery, Tehran University of Medical Sciences, Tehran, Iran; 4 Department of Biostatistics, School of Nursing and Midwifery, Iran University of Medical Sciences, Tehran, Iran

**Keywords:** Honey Syrup, Pain, Nulliparous women

## Abstract

**Background:**

Labor is a natural pain despite the fact that the severity of the pain and response to it differ in various people, but most women refer to labor as an unpleasant experience of their lives. The present study was carried out to determine the effect of honey on the severity of labor in primigravida women.

**Methods:**

In this study, 80 healthy volunteer primigravida women were entered to the research as randomized clinical trial (40 subjects in each group) after studying and signing consent form and they were randomly divided into two intervention honey group and control group. The data gathering tool comprised four main parts of the personal profile questionnaire, controls performance in the labor, records fluid intake rate and pain intensity using a 0-10 cm ruler.

**Results:**

The mean of pain intensity in the honey syrup intervention group was significant.

**Conclusion:**

Honey syrup consumption without side effects significantly affected the severity of labor.

The experience of parturition is a unique event in a woman's life, which has long been portrayed as a painful event. The severity of this pain is greater than the pain of arthritis, toothache, fracture, postoperative neuralgia, phantom pain, cancer pain and chronic pain in the lumbar; also, they are severe in primigravida women than multigravida women (1). Today, at higher levels, control and reduction of labor are considered as the basic rights of individuals. For this reason, the methods of labor and pleasure of parturition process are very important. Methods of labor reduction include alternative medicine and complementary medicine (2). In the past, the advocates of limitation of receiving fluids and food in labor, which was based on Cortez Mendelssohn (1940) theory of bronchopulmonary response followed by aspiration of stomach contents, were opposed to nutrition during labor. Given that the WHO has called the countries with a history of traditional medicine to return to traditional medicine (3), the research team decided to use honey, which is a traditional and indigenous nutrient in Iran, to assess its impacts on the labor of primigravida women. Among the medical effects mentioned about honey are: its antibacterial effects, antiviral, anti-cancer, anti-oxidant properties, treatment of infectious wounds, skin diseases and burn remediation (4). In addition, some studies have investigated the effects of honey on reducing severe pain in menstruation, pain after cesarean section, pain after tonsillectomy and also the use of royal jelly from honey products had a dramatic effect on the physical pains of premenstrual syndrome (5).

Therefore, the research group decided to conduct a study by performing an intervention of honey syrup on labor pain of primigravida women and present necessary recommendations in the case of achieving pain reduction after taking this nutrient. 

## Methods

In this randomized clinical trial, 80 volunteer primigravida women eligible for inclusion (gestational age 38-42 weeks, cephalic presentation, physical and mental health, no history of infertility, having a normal delivery conditions, opening the cervix at a rate of 4-7 cm, having no history of allergy to honey), were randomly placed into one of the honey syrup intervention group and control group in the active phase of labor with dilatation of 4-7.

Based on previous studies in similar fields and with regard to the confidence level of 90% and 80% power and with the possibility of a 10% drop in the sampling, the final sample size of 40 patients in each group (total of 80) using this formula was calculated.

$\frac{1}{1-f}\times \frac{2{\left(Z_{1-\frac{\propto }{2}}+Z_{1-\beta }\right)}^{2}S^{2}}{{\left(\mu_{1-\mathrm{\mu 2}}\right)}^{2}}$

In this study, samples were randomly assigned to the honey syrup group (Card No. 1( or placebo group (card No.2). The first person who chose a card was randomly selected, and then placed in each of the two groups and the next person was placed in the other group. For example, if the first person chose number two, he would be placed in the control group, and as a result, the next person would be in the honey syrup group, and again for the third one, both cards were submitted for selection. Also, none of the samples were aware of the nature of the numbers 1 and 2 cards, and after the selection of the card, the process of doing research was explained to them. The random allocation of samples was done by the researcher, but to prevent bias in the research, the researcher's colleague (who was not aware of the nature of each person in each of the two groups) was used to record the severity of pain.

Given that one-on-one care during the labor process could have an effect on mental health, to unify the interventional factor in both the intervention group and the control group, the researcher decided to stay close to the patient during the entire active phase of childbirth. In this research, the methods of data collection were divided into four main sections: personal characteristics questionnaire (age, education, employment status, current pregnancy tendency, gestational age, abortion history), registration of controls in labor (vaginal examinations, length of contractions, distance of contractions and heart rate of the fetus), record the intake amount of fluids (date syrup or water) and record pain intensity using a 0-10 cm ruler. 

In the entire research process, the researcher conducted the control and registration of all examinations in two intervention and control groups, so that the length and intervals of the uterine contractions and the number of heartbeat of the fetus at the time of entry, then every 30 minutes and dilatation, efasman, station, the condition of the water bag at the time of entering the study, and then every 2 hours were assessed by performing a vaginal examination according to the national protocol. Also, one by one support by the researcher, despite completing the research process which is up to 8 cm dilatation, was continued until the end of delivery stage because it was morally wrong to stop the withdrawal of support. In order to prevent bereavement during the study, the pain severity was recorded by a research fellow using a pain ruler, so that at the start of the study, then every 30 minutes, a research fellow using a 0-10 cm ruler and after training the samples, asked them about the severity of pain and recorded them.

Since the study was double-blind, the participants and research fellow were blind to the intervention, as well as the statistician until the analysis was completed. To intervene with honey syrup after a large study of the relevant sources, an amount of 2.5 teaspoons of honey of a product of one of the standard certified factories with the standard mark registered on its container (for all the same specimens) was mixed with 150 ml of water. There was no intervention in the control group. Providing the above fluids at the beginning of the study and then every 30-60 minutes and the volume of the received fluids with the time of receipt were registered. In the control group, simple water was given and recorded. 

## Results

 The type of tests used for demographic characteristics and vaginal examinations has been entered in table 1. 

Non-matching of pain in the honey syrup group and control group at the beginning of the study could influence the interpretation of results. Therefore, pain severity was determined before intervention and the results of ANOVA showed that the acquired mean score of pain in both groups was not significantly different and both groups were in the same level at the beginning of the study in terms of pain intensity (P=0.44). ANOVA was used at 30 and 150 minutes after the intervention to compare the pain intensity. Results showed that there was a significant difference between the two groups after 30 minutes after intervention (P=0.028). There was a statistically significant difference between the two groups at 60 minutes after intervention and consumption of honey syrup significantly decreased pain (P=0.000). In the 90 minutes, the use of honey syrup significantly reduced the pain compared to the control group (p=0.003). Also, in the 120 minutes, the use of honey syrup significantly reduced the pain compared to the control group (P=0.022). But it was not significantly reduced in 150 minutes after intervention of honey syrup group compared to the control group (table 2).

**Table 1:** Comparison of the frequency of vaginal examination and demographic characteristics of subjects at baseline

**Characteristics **	**Honey syrup** **N=40**	**Control** **N=40**	**Test**	**P value**	**Partial Eta Squared**
Age (year), Mean±SD	25.65± 4.12	24.90±4.21	ANOVA	0.789	0.001
Education (diploma), n (%)	16 (40)	15 (37.5)	X^2^	0.898	0.001
Occupation (housewife), n (%)	36 (90)	36 (90)	X^2^	1.00	0.00
Tend to pregnancy: demands, n (%)	39 (97.5)	39 (97.5)	X^2^	1.00	0.00
Gestational age (weeks) Mean(SD)	39. 4±0.99	39.65±1.00	ANOVA	0.320	0. 013

**Table 2:** Comparison of pain intensity in two groups with VAS

**Time**	**Honey syrup** **Mean(SD)**	**Control** **Mean(SD)**	**P-value**	**Partial Eta Squared**
Beginning of study	8.35±1.66	8.19±1.84	0.44	0.008
30 minutes after intervention	7.20±1.63	8.17±1.72	0.028	0.179
60 minutes after intervention	7.62±1.58	9.05±1.08	0.0001	0.321
90 minutes after intervention	8.84±1.05	9.58±0.78	0.003	0.243
120 minutes after intervention	9.37±0.92	9.80±0.53	0.022	0.186
150 minutes after intervention	9.75±0.7	9.91±0.41	0.426	0.021

## Discussion

This study was carried out to evaluate the analgesic effects of honey on labor pain intensity of primigravida women. The findings of this study showed that honey consumption significantly reduces labor pain. In the same vein, in a study conducted by Mirbagher & Aghajani (2013), the severity of menstrual pain in honey users was significantly reduced in the first three hours after the beginning of menstruation, while the menstrual pain was not reduced after three hours.

The results of this research are almost in line with the results of our research. In our research, the pain was significantly reduced immediately after honey consumption up to 120 minutes after intervention compared to the control group but after 150 minutes, the labor pain has not occurred. In the study of Aghajani, the analgesic effects of honey are related to the impact of honey on the reduction of prostaglandin concentration in the blood (6). In a study by Ergol et al (2012) aimed at investigating the effect of oral nutritional intake on labor development and labor pain, grape juice was used for intervention, and the results indicate that the pain intensity of the active phase of delivery that was measured using VAS, was not significantly different in the oral intake of grape juice group compared to the control group (P=0.468) (7), the obtained results are not consistent with the results of present research. The study of Hikmatzadeh et al (2011) showed that following an oral ingestion of dill seed brew, the severity of labor pain in the same dilatations was significantly lower in the intervention group than the control group. In our study, considering the increasing nature of labor pain, the severity of labor pain was evaluated every 30 minutes using pain ruler and this measurement was not evaluated in the same dilations (8). In the study, the presence of phenolic compounds in the dill seed has been emphasized, and honey also contains phenolic compounds, which affects smooth muscle contractions, which is due to labor pain following uterine contractions. Therefore, phenol-containing compounds may also affect labor pain.

A study by Schipper et al. (2002), aimed at examining the effect of oral intake of carbohydrate-based liquids on the effects of labor, the criteria for the use of analgesic drugs including pethidine, entonox and epidural were used in order to evaluate the pain level in the units. There was no significant difference between the two groups in carbohydrate intake and the control group, which implies that carbohydrate intake has not reduced the use of painkillers (9). 

It should be noted that in the studies of Rahmani and Scheepers, the pain parameter was not evaluated using the appropriate pain intensity tool, such as the visual scale of pain, and only the rate of analgesia was studied. In both studies, the rate of analgesia between the intervention and control groups was not significant. 

Therefore, due to the limited resources and contradictory results from existing research, further studies seem necessary in this field. One of the limitations of this study was the noise, crowding and movement of people in the labor room, which could have an impact on the management of labor pain and childbirth. Therefore, to control this issue, the samples were studied in the independent unit of labor room in the hospital.  
